# Retrospective Review of Missed Cancer Detection and Its Mammography Findings with Artificial-Intelligence-Based, Computer-Aided Diagnosis

**DOI:** 10.3390/diagnostics12020387

**Published:** 2022-02-02

**Authors:** Ga Eun Park, Bong Joo Kang, Sung Hun Kim, Jeongmin Lee

**Affiliations:** Department of Radiology, College of Medicine, Seoul Saint Mary’s Hospital, The Catholic University of Korea, Seoul 06591, Korea; hoonhoony@naver.com (G.E.P.); rad-ksh@catholic.ac.kr (S.H.K.); jmlee328@gmail.com (J.L.)

**Keywords:** breast cancer, computer-aided diagnosis, artificial intelligence, mammography

## Abstract

To investigate whether artificial-intelligence-based, computer-aided diagnosis (AI-CAD) could facilitate the detection of missed cancer on digital mammography, a total of 204 women diagnosed with breast cancer with diagnostic (present) and prior mammograms between 2018 and 2020 were included in this study. Two breast radiologists reviewed the mammographic features and classified them into true negative, minimal sign or missed cancer. They analyzed the AI-CAD results with an abnormality score and assessed whether the AI-CAD correctly localized the known cancer sites. Of the 204 cases, 137 were classified as true negative, 33 as minimal signs, and 34 as missed cancer. The sensitivity, specificity and diagnostic accuracy of AI-CAD were 84.7%, 91.5% and 86.3% on diagnostic mammogram and 67.2%, 91.2% and 83.38% on prior mammogram, respectively. The AI-CAD correctly localized 27 cases from 34 missed cancers on prior mammograms. The findings in the preceding mammography of AI-CAD-detected missed cancer were common in the order of calcifications, focal asymmetry and asymmetry. Asymmetry was the most common finding among the seven cases, which could not be detected by AI-CAD in the missed cases (5/7). The assistance of AI-CAD can be helpful in the early detection of breast cancer in mammography screenings.

## 1. Introduction

Mammography is proven to be an effective method for reducing the mortality of breast cancer [1]. However, mammography has inherent limitations. Factors that contribute to lowering the sensitivity of mammography are dense breast parenchyma, rapid tumor growth rate, and the finding and reading of subtle errors (perceptual or interpretive). Studies have shown that approximately one-third of newly diagnosed breast cancers were retrospectively visible in prior mammograms [2,3].

Missed cancer refers to cancer that can be retrospectively visualized in preceding mammograms that were initially interpreted as negative. The use of the term “missed” should not be construed as implying negligence in interpretation because the judgment of lesion visibility is made only in retrospect [4]. Missed cancer can be classified as false-interval cancer, subsequent screen-detected cancer and alternative-imaging-detected cancer. Suggested methods to reduce the occurrence of missed cancer include additional supplementary images, improved image quality and interpretation techniques, double reading, and computer-aided detection (CAD) [5].

Recent studies showed that the performance of artificial intelligence-based computer-aided detection (AI-CAD) for mammography was non-inferior, or even superior to that of radiologists and could be a reliable decision support tool. This AI-based mammography reading is thought to have the potential to improve missed cancer detection by particularly reducing perceptual and interpretive errors [6,7,8,9]. Prior studies showed that AI-CAD improved the detection of missed cancer in prior mammography [10,11].

## 2. Materials and Methods

### 2.1. Study Population

This retrospective study was approved by the Institutional Review Board (IRB) and informed consent was waived. Among the patients diagnosed with biopsy-proven malignancy in this hospital between 2018 and 2020, 263 patients with diagnostic and prior mammograms within 36 months were enrolled. A total of 204 patients were included, excluding prior breast cancer surgery on the ipsilateral breast (*n* = 47), recognition error by AI-CAD (*n* = 7), and import failure (*n* = 5).

### 2.2. Imaging Analysis

The retrospective mammography review was performed in consensus by two breast imaging specialists with 16 and 4 years of experience, respectively. The cancers were classified as true negative, minimal signs, or missed cancer based on the findings from prior and diagnostic (present) mammograms. True negative refers to no evidence of cancer on prior mammograms in retrospective reviews. Minimal signs refer to subtle abnormality which would not necessarily be regarded as warranting assessment on a prior mammogram [12,13]. Mammographic findings were described as mass, mass with calcifications, calcifications, asymmetry, focal asymmetry and architectural distortion. Breast density was determined by consensus of two readers based on the Breast Imaging Reporting and Data System (BI-RADS) 5th edition. In addition, clinical information of missed cancer such as final pathology, IHC (immunohistochemistry) type, and TNM stage was collected via medical records.

### 2.3. Imaging Analysis by AI-CAD

A commercial AI-CAD software (Lunit INSIGHT for Mammography, v1.1.4.3, Lunit Inc., Seoul, Korea, available at https://insight.lunit.io, accessed on 7 December 2021) dedicated to breast cancer detection and diagnosis on digital mammography was used. This AI-CAD was developed with deep convolutional neural networks (CNNs), trained, and validated through multi-national studies with over 170,000 mammography examinations [8,14,15]. This AI-CAD software presented its results as separate gray-scale images that contained an overall per-breast abnormality score for each CC (craniocaudal) and MLO (mediolateral oblique) image, and a gray-scale heatmap that marked areas of abnormality using a line of varying thickness to indicate the probability of malignancy (POM). The abnormality score is provided in percentages of 0–100%; less than 10% is presented as “low” and does not appear as a separate result. When more than one area is detected, the highest abnormality score is provided at the bottom as a result.

Two radiologists determined whether the AI-CAD correctly localized the known malignant lesion in diagnostic and prior mammograms. If matched, the higher score from CC or MLO view was recorded. False positive was defined as follows: (a) When AI-CAD evaluates a negative mammography by radiologists as abnormal and (b) when the area marked by AI-CAD with the highest abnormality score does not match the known malignant lesion.

### 2.4. Statistical Analysis

Diagnostic performance of AI-CAD was evaluated with sensitivity, specificity and diagnostic accuracy. The correlation of classified groups in relation to abnormality score by AI-CAD was analyzed with the Kruskal–Wallis test. The comparison of abnormality scores among the different classification groups was performed with a post hoc Bonferroni correction for multiple comparisons. The significance threshold was set at 0.05. All calculations were performed using SPSS software (version 21, SPSS Inc., Chicago, IL, USA). A *p*-value of less than 0.05 was considered to indicate statistical significance.

## 3. Results

### 3.1. Patient Characteristics

The patient characteristics are summarized in Table 1. The mean age of the included patients was 53.9 years (range 25–84). The mean interval duration between diagnostic and prior mammograms was 23.8 months (range 6–36). Mammographic breast parenchymal density was categorized as almost entirely fat in 3 cases (1.5%), scattered fibroglandular tissue in 42 cases (19.6%), heterogeneously dense in 94 cases (47.1%), and extremely dense in 65 cases (31.9%). The dense breast rate was 78.9%.

### 3.2. Mammography Classification Results by Radiologists

Two radiologists classified the included 204 cases as true negative (*n* = 137), minimal signs (*n* = 33) and missed cancer (*n* = 34) in consensus. Of the 137 true negative cases, 90 cases were visible and 47 cases were not visible (occult) on diagnostic mammograms. Overall, 157 cases were mammography-visible on diagnostic mammograms, and 67 cases were visible on prior mammograms. The dense breast rate was 83.2% (114/137) in the true negative, 78.8% (26/33) in minimal signs and 61.8% (21/34) in missed cancer groups.

### 3.3. Mammography Findings

**Figure 1:**
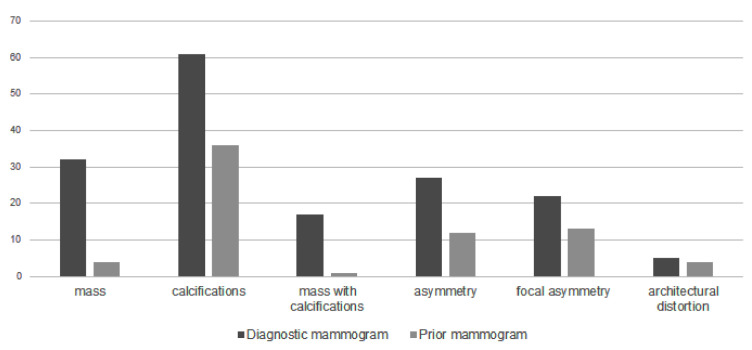
Mammographic features on diagnostic and prior mammograms.

Figure 1 shows the distribution of mammographic findings on diagnostic and prior mammograms. Calcifications, mass, asymmetry, focal asymmetry, mass with calcifications and architectural distortion were common in the order of diagnostic mammograms. The proportion of calcification, asymmetry and focal asymmetry was high in prior mammograms, while the proportion of mass and mass with calcifications increased in diagnostic mammograms.

### 3.4. AI-CAD Results

**Figure 2:**
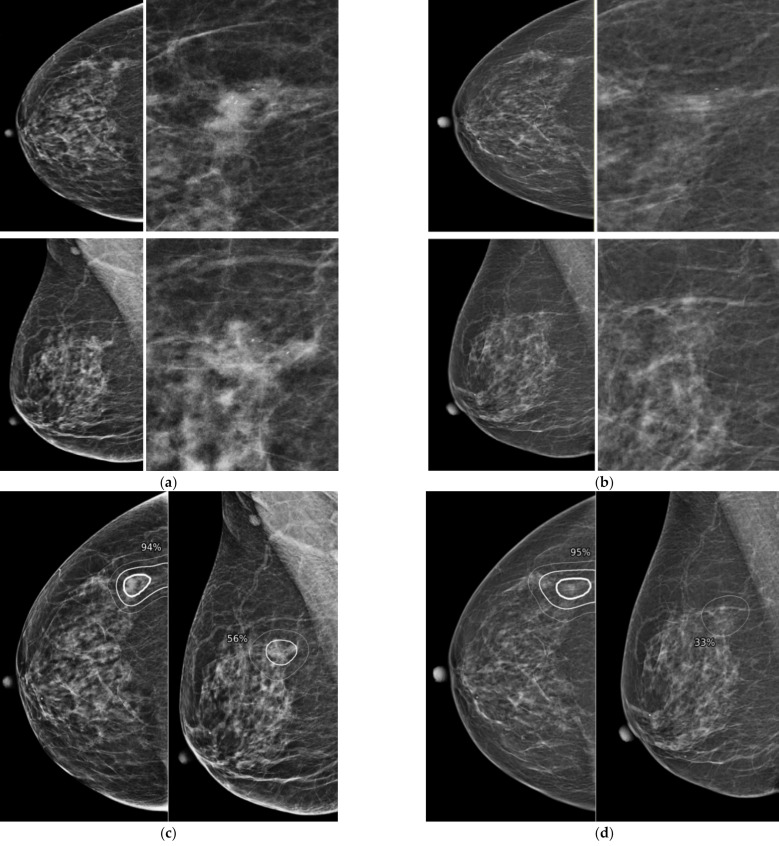
Representative case of missed cancer detected by AI-CAD. A 67-year-old woman had a focal asymmetry with increased number of calcifications in diagnostic mammogram (**a**). After biopsy, this lesion was confirmed as invasive carcinoma. When the two radiologists reviewed the prior mammogram performed 12 months ago (**b**), asymmetry visible on CC view and several calcifications were retrospectively detected at the same location. When the AI-CAD was retrospectively applied, the AI-CAD identified the exact location of lesion in the diagnostic mammogram (**c**) and prior mammogram (**d**).

Table 2 represents the AI-CAD results for diagnostic and prior mammograms. The AI-CAD correctly localized 27 of 34 missed cancer (Figure 2) and 18 of 33 minimal signs on prior mammogram. The false positive rate in prior mammograms was 5.8% (12/204). The overall sensitivity, specificity and diagnostic accuracy of AI-CAD were 84.7%, 91.5% and 86.3% in diagnostic mammograms and 67.2%, 91.2%, 83.3% in prior mammogram (Table 3).

### 3.5. Missed Cancer Detected by AI-CAD

**Figure 3:**
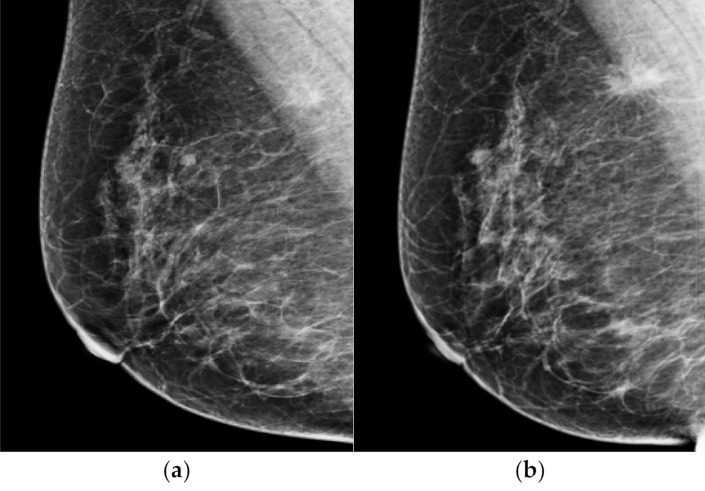
Representative dismissed case by AI-CAD. A 62-year-old woman classified as negative in a mammography screening (**a**). When looking at the mammography in retrospect, asymmetry only seen in MLO view was newly developed in the upper and posterior aspect of right breast. This lesion was missed in the prior mammogram. When analyzed retrospectively, AI did not recognize this lesion either. (**b**) After 18 months of diagnostic mammogram, the previous asymmetry became a spiculated mass and a biopsy confirmed it as invasive cancer.

The AI-CAD did not detect suspicious findings in 7 of the 34 missed cancer on prior mammogram. Of the seven cases, the most common finding was asymmetry (*n* = 5) (Figure 3), and the other was focal asymmetry (*n* = 2). All undetected lesions were isodense in mammograms. These lesions were located in the parenchyma (*n* = 3), the retromammary fat layer (*n* = 3), and the premammary fat layer (*n* = 1). All five asymmetries were only visible on MLO view.

**Figure 4:**
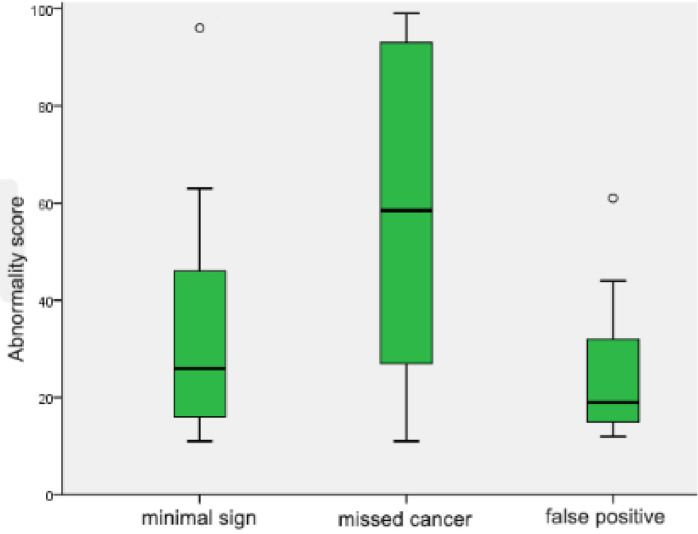
Comparison of abnormality scores in prior mammograms. Box and whiskers plot showing the distribution of abnormality scores of minimal signs, missed cancer and false positive cases. All box plots proved the median value (solid line), interquartile range (green box), 95% confidence interval (black whiskers) and outliers beyond the 95% confidence interval (blank circles).

Figure 4 shows the comparison of abnormality scores between groups on prior mammogram. The median value (interquartile range (IQR)) of the abnormality score was 26 (17, 45.8) for minimal signs, 58.5 (28, 91.3) for missed cancer, and 19 (15, 32) for false positive cases. There was a significant difference in abnormality scores between missed cancer and minimal signs (*p* = 0.042); and missed cancer and false positive cases (*p* = 0.027). However, there was no significant difference between minimal signs and false positive cases (*p* > 0.05).

### 3.6. Characteristics of Missed Cancers

Table 4 represents the characteristics of missed cancer that AI-CAD correctly localized in prior mammograms. The frequent mammography findings were in the order of calcifications, focal asymmetry, asymmetry, architectural distortion, and mass with calcification. Most of the cases were ER (estrogen receptor)-positive (23/27). The IHC types of 27 cases were as follows: 16 luminal A cases, 7 luminal B cases, 2 HER2-enriched cases and 2 TNBC cases. For the final pathology, seven cases were ductal carcinoma in situ (DCIS), 20 cases were invasive cancer and five cases were lymph-node-positive. The distribution of the stages was as follows: Stage 0 (7/27), stage I (11/27), stage II (8/27) and stage IV (1/27). Stage IV patients were diagnosed with bone metastasis at the time of diagnosis.

## 4. Discussion

The aim of this retrospective study was to assess the potential of using AI-CAD to improve the detection of missed cancer in mammography screenings. We classified the included cases via retrospective reviews of diagnostic and prior mammograms, and 32.8% of these were false negative (minimal signs and missed cancer: 67/207). Our classification results were similar to the results from previous studies. Depending on the review methods, it is reported that 10 to 30% of all interval cancers and 25 to 40% of screen-detected cancers are classified as false negative in retrospect [2,3]. False negative cases were subcategorized into missed cancer and minimal signs in this study. This is because unnecessary recall would be greatly increased, despite the fact that false negatives can be reduced if we include all minimal signs by lowering the threshold in clinical practice. [16]. Even if a case with minimal signs is recalled, it may not necessarily lead to the diagnosis of breast cancer [17].

AI-CAD correctly identified 27 of 34 missed cancer (79%) in prior mammogram. In addition, AI-CAD showed a high accuracy (86.3%) in diagnostic mammograms and a high specificity (91.5%) in prior mammograms. The false positive rate was 5.8%. The abnormality score of missed cancer was significantly higher than that of minimal signs and false positive groups in prior mammograms (Figure 4). This result suggests that false negative cases were appropriately classified into two groups: minimal signs and missed cancer. It also suggests that false positive results would not interfere with the early detection of missed cancer with AI-CAD. However, the clinical implication of the abnormality score provided by the AI-CAD has not yet been fully elucidated.

The common mammography findings in missed cancer included calcification, asymmetry, and focal asymmetry. However, mass was the most common finding in previous studies [18,19]. Of the included patients, 83.2% of true negative, 78.8% of minimal signs and 61.8% of missed cancer had dense breast. The missed cancer group had a relatively low percentage of dense breast compared to the other groups. This implies that the perception and interpretative errors that lead to missed cancer may not be deeply related to breast density. A previous study also showed that an increase in breast density contributed to lowering the sensitivity; however, there was no significant difference in specificity [20].

In this study, the AI-CAD found that all five cases of missed cancer showed an architectural distortion in prior mammograms. In one case, architectural distortion was missed and developed into stage IV breast cancer 10 months later. Architectural distortion is known to be the most commonly missed abnormality in false negatives, and one study showed that 45% (9/20) of missed findings were due to architectural distortion [21].

Most of the missed cancers detected by AI-CAD were early-stage (26/27) and ER-positive (23/27). Among the IHC types, luminal A was the most common in 16 patients (59.3%). Hovda et al. reported that the estrogen receptor positivity was 95% (215/234) in missed cases [19]. Kim et al. reported that the most common presentation in both screening and symptomatic groups was luminal A (63.6% and 54.3%, respectively) [22].

The AI-CAD proved an excellent detection rate, yet it was not able to detect all abnormalities. The most common finding that AI-CAD was not able to detect was asymmetry. As shown in Figure 3, the asymmetry noted in prior mammograms was a newly developed lesion. Radiologists have the advantage of being able to compare current images with previous images more freely and are able to make decisions through correlations between CC and MLO views, and between mammograms and other imaging modalities. Deep-learning-based AI was developed and received a lot of attention. However, studies have shown that it is not enough to replace the role of radiologists. This is because the reading process is not just a detection of abnormality, but a more comprehensive process of judgement, consideration and communication [23,24]. Reading mammography is still challenging. The role of radiologists is also important, and the aid of AI-CAD will help reduce the burden of the reading process.

There are several limitations in this study. First, this retrospective study included only a small number of patients with biopsy-proven malignancy. Thus, selection bias was inevitable. Second, only a single AI-CAD software was used for analysis. Future updated versions or other AI-CADs may show different results from this study. In addition, it is still difficult to determine the extent to which the suspicious findings detected by the AI-CAD in prior mammograms will lead to early cancer detection in actual practice. Additionally, false positive findings can affect the radiologist’s judgment and lead to an increase in recall rate. A further assessment in a prospective design with a larger number of patients will be required for the implications of the AI-CAD in mammography screening.

In conclusion, this retrospective study showed that the assistance of AI-CAD has the potential to facilitate early cancer diagnosis.

## Figures and Tables

**Table 1:** Baseline patient characteristics.

	Patient Number (%)
Age	
50<	88 (43.14%)
50≥	116 (56.86%)
Breast density	
(a) almost fatty	3 (1.5%)
(b) scattered	40 (19.6%)
(c) heterogeneous	96 (47.1%)
(d) extreme dense	65 (31.9%)
Interval ^†^	
0–12 months	83 (40.7%)
13–36 months	121 (59.3%)

^†^ Interval = months between diagnostic and prior mammograms.

**Table 2:** AI-CAD results for diagnostic and prior mammograms.

AI-CAD Result	Diagnostic Mammogram	Prior Mammogram		
		True Negative	Minimal	Missed
True positive	133	0	18	27
True negative	43	125	0	0
False positive	4	12	0	0
False negative	24	0	15	7
Total	204	137	33	34

**Table 3:** Diagnostic performance of AI-CAD.

	Diagnostic Mammogram	Prior Mammogram
Sensitivity	84.7% (133/157)	67.2% (45/67)
Specificity	91.5% (43/47)	91.2% (125/137)
Accuracy	86.3% (176/204)	83.3% (170/204)
PPV	97.1% (133/137)	78.9% (45/57)
NPV	64.2% (43/67)	85.0% (125/147)

PPV (positive predictive value), NPV (negative predictive value).

**Table 4:** Mammography findings of missed cancer in prior mammograms.

	Mammography Finding	IHC Type	T	N	Stage
1	Architectural distortion, Calcifications	TNBC	2	0	IIA
2	Calcifications	Luminal B	is	0	0
3	Architectural distortion	Luminal A	1a	1	IIA
4	Calcifications	Luminal A	1mic	0	IA
5	Focal asymmetry	Luminal A	1c	0	IA
6	Architectural distortion	Luminal A	1a	0	IA
7	Calcifications	HER2-enriched	is	0	0
8	Architectural distortion	Luminal A	1a	0	IA
9	Asymmetry	TNBC	2	0	IIA
10	Calcifications	Luminal A	is	0	0
11	Focal asymmetry	Luminal A	1a	0	IA
12	Focal asymmetry	Luminal A	1c	0	IA
13	Focal asymmetry	Luminal A	1c	0	IA
14	Calcifications	Luminal A	is	0	0
15	Calcifications	Luminal B	1c	0	IA
16	Calcifications	Luminal A	is	0	0
17	Asymmetry	Luminal A	1c	0	IA
18	Focal asymmetry	Luminal B	2	0	II
19	Calcifications	HER2-enriched	is	0	0
20	Calcifications	Luminal A	1c	0	IA
21	Focal asymmetry, Calcifications	Luminal A	2	0	IIA
22	Focal asymmetry, Calcifications	Luminal A	2	1	IIB
23	Focal asymmetry	Luminal B	2	1	IIB
24	Calcifications	Luminal B	is	0	0
25	Mass with calcifications	Luminal B	2	1	IIB
26	Calcifications	Luminal A	1a	0	IA
27	Architectural distortion	Luminal B	2	0	IV

## Data Availability

All data generated and analyzed during this study are included in this published article. Raw data supporting the findings of this study are available from the corresponding author on request.
